# Cultural Variance in Reception and Interpretation of Social Media COVID-19 Disinformation in French-Speaking Regions

**DOI:** 10.3390/ijerph182312624

**Published:** 2021-11-30

**Authors:** Brian Hughes, Kesa White, Jennifer West, Meili Criezis, Cindy Zhou, Sarah Bartholomew

**Affiliations:** 1 Polarization and Extremism Research and Innovation Lab (PERIL), American University, Washington, DC 20016, USA; kesa@american.edu (K.W.); JW2433a@student.american.edu (J.W.); criezis@american.edu (M.C.); czhou@american.edu (C.Z.); sb2468b@student.american.edu (S.B.); 2 Program of Justice, Law, and Criminology, School of Public Affairs, American University, Washington, DC 20016, USA

**Keywords:** misinformation, disinformation, conspiracy theories, COVID-19, qualitative coding, francophone, social media, persuasion

## Abstract

Digital communication technology has created a world in which media are capable of crossing national boundaries as never before. As a result, language is increasingly the salient category determining individuals’ media consumption. Today, a single social media post can travel around the world, reaching anyone who speaks its language. This poses significant challenges to combatting the spread of disinformation, as an ever-growing pool of disinformation purveyors reach audiences larger than ever before. This dynamic is complicated, however, by the diversity of audience interpretations of message content within a particular language group. Both across and within national boundaries, a single message may be subject to a variety of interpretations depending on the cultural experiences and identities of its recipients. This study explores that dynamic through analysis of French language anti-vaccine and COVID-denialist disinformation. Using qualitative coding methodology, a team of researchers empirically identify common and far-reaching patterns of Francophone COVID disinformation narratives and rhetoric. These narratives and rhetorics are then subjected to hermeneutic close reading to determine likely variations in their reception across different French-speaking cultures. Data were gathered and analyzed between the dates of 24 March 2021 and 27 April 2021. Results of this study indicate the need for awareness on the part of public health officials combatting COVID disinformation online, for both the transnational reach of disinformation targeting speakers of a single language and for variations in meaning and salience across cultures within that language group.

## 1. Introduction

This study seeks to identify and analyze some of the most common narrative tropes and rhetorical strategies found in French-language antivaccine and COVID-denialist social media and user-generated content.

### 1.1. Vaccine Misinformation and Disinformation Online

Misinformation, disinformation, and malinformation refer to varieties of untrue or misleading messages. This study grounds itself in a pragmatic and inclusive theory of the problematic, such as that described by Tucker et al. for the Hewlett Foundation [1]. This perspective stands on several founding principles, namely, that mis-, dis-, and malinformation are detrimental to democratic policymaking [1,2,3]; that key affordances of online media such as low barrier to entry, anonymity, and affective polarization all contribute to a decline in quality of political and other policy discussions and an increase in the quantity of mis-, dis-, and malinformation [1,4,5,6]; and that a diverse array of actors, from the individual to the grassroots, government to the automated—each with their own methods and motives—contribute to the production of an online ecosystem brimming with misinformation, disinformation, and malinformation [1,4,7]. This study also acknowledges that misinformation and disinformation can be difficult to distinguish, as the difference rests on a question of the purveyors’ motive [8,9].

In the context of the COVID-19 pandemic, misinformation and disinformation can be defined as messages that contradict the best-available expert information and advice relating to public health and its attendant political, economic, and social conditions [10,11]. “Misinformation” is defined by its unintentional inaccuracy, while “disinformation” is knowingly fabricated and shared with the intent to mislead toward some ulterior motive [12,13]. Malinformation is distinguished by being agnostic to issues of facticity; it may or may not be true, yet it is specifically deployed in ways and contexts intended to mislead or harm [14]. All three forms can originate from citizens, governments, and foreign bodies. Their motives can be malign or benign, based in earnest ignorance or a cynical strategic program of harm. Whatever the motive, throughout the COVID-19 disease and pandemic, fabrication and propagation of unsubstantiated claims have steadily increased, creating a parallel public health information crisis, which some have termed the “infodemic” [14,15,16,17].

Mis-, dis-, and malinformation frequently take the form of “fake news,” a flexible term that refers to non-factitious or misleadingly factitious communication presented as news [18,19]. The digital age has created a news environment highly conducive to the spread of misinformation and disinformation, particularly as “citizen journalism challenged the link between news and journalists [and] social media offered a wider platform for non-journalists to engage in journalism” [19] (p. 139). Tadoc et al. point out how digitization, and social media in particular, have changed expectations as to the format and appearance of legitimate news, both in terms of the news message and its means of distribution.

Online fora are demonstrated to be a key contributor to the spread of vaccine hesitancy [20,21,22,23,24]. Access to digital communication technology has spread mis-, dis-, and malinformation through decentralized networks [4]. Social media platforms in particular appear to play a central role, both before and during the COVID-19 pandemic [25,26,27,28]. The digital francophone world comprises a relatively tight network connecting online media between France and other French-speaking countries, particularly in Africa [29]. This has led to vaccine hesitancy content originating in France to permeate networks in Francophone Africa [30]. False information regarding chloroquine as a COVID-19 treatment has similarly diffused from France to other Francophone countries, leading to widespread self-medication with chloroquine as a COVID-19 preventative and treatment, and in turn leading to many cases of overdose [31]. Francophone African countries have served as their own incubators of viral misinformation and disinformation, including popular apocalyptic conspiracy theories [29]. And outlets throughout the French-speaking world act as “info-laundering” devices, translating Russian and U.S. misinformation, disinformation, malinformation, and conspiracy theories relating to the COVID-19 pandemic and disseminating them throughout the francophone digital network [32].

### 1.2. National vs. Linguistic Borders and Online Communication

For the last decade, observers have debated as to the “post-Westphalian” or “Cyber Westphalian” quality of the internet [33,34,35,36], debating the effects of networked digital communication on the salience of national identities and state borders. To date, pronouncements as to the nation-state’s digital age obsolescence or its ruthless self-reassertion have proven overstated. National governments increasingly seek to assert sovereignty over digital space [37,38,39], while media industries seek on one hand to cultivate international audiences while protecting their domestic market advantages [40,41]. However, it cannot be denied that despite government interference and the still-prevalent “digital divide,” today’s audiences have far greater access to foreign media, both commercial and social, than ever before in human history [42,43,44].

So while state control of telecommunication infrastructure remains intact, and in many cases even expanding, digital communication continues to spread across state borders. If the inner limit of digital communication is access (state censorship and/or the “digital divide”), then the outer limit of online communication is less state regulation than legibility—that is, language [45,46,47]. The internet has been a powerful force for multilingualism, despite early expectations that it would lead to further global “Englishization” [48]. Hafez argues that language represents one of the critical barriers to the emergence of a true, global, public sphere [49]. “Even where media technologies and formats are explicitly transnational, as with satellite and cable TV, considerable adaptation to local and regional languages, cultures, and expectations concerning program genres has been a condition of viability for operating in different countries” [50] (p. 627). As Taneja and Wu succinctly put it: “websites cluster according to language and geography” [39] (p. 297). Yet despite digital media’s transnational reach, local circumstances prohibit the cultural flattening predicted by early techno-optimists and cheerleaders of globalization [51]. Perhaps even because of the porousness of national boundaries with respect to communication, national and local identities have stood fast, and in some cases re-entrenched [52,53].

This points to a situation in which (1) communication flows widely (but not unfettered) across state borders, which is (2) limited largely (but not exclusively) by linguistic intelligibility, to (3) audiences that may share a language but do not necessarily share a culture or identity. Under such circumstances the meaning imputed to digital content by its audience can be extremely varied. This phenomenon can perhaps be best modeled using the “encoding/decoding” model of communication first articulated by Stuart Hall [54]. In this model, Hall proposed that a message’s intended meaning and its received meaning “may not be perfectly symmetrical” [54] (p. 260). Hall proposed that a message might be understood at the valence of its producer (the “dominant or hegemonic” interpretation), the ideological functionaries of that producer (the “professional” interpretation), critically (a “negotiated” interpretation), or counter-hegemonically (an “oppositional” interpretation) [54] (pp. 272–274). Hall theorized this model prior to the age of social media and the digital prosumer. Today “elite” media production is no longer so evident, as new media forms such as memes, message boards, quote-Tweets etc. represent what Nowak terms “multiparticipant online content” [55]. However, Hall’s influential theory offers a ground from which to understand the simultaneous coexistence of many different interpretations and understandings of the same message. This theoretical perspective is useful in analyzing the ways in which a single piece of content might carry with it myriad meanings, some parallel and some contradictory, depending on its audience and their circumstances.

### 1.3. Persuasive Messaging: Targeting Narratives & Rhetoric vs. Targeting Facts

This codebook was developed as part of a toolkit of background materials supporting public health message campaigns in favor of COVID vaccination and against COVID-denialism and other forms of public health misinformation and disinformation. The field of pro-social persuasion reaches across numerous domains, from public health to preventing violent extremism [56,57,58]. And throughout the literature of these various domains, certain patterns emerge pertaining to the efficacy of fact- and logic-based persuasion campaigns versus campaigns based in emotion, identity, narrative/rhetoric and values. While the literature is not uniform, there appears to be strong evidence suggesting that persuasive campaigns focusing on message form (i.e., emotion, identity, narrative/rhetoric, values, etc.) tend to be more broadly successful than those focusing on logic and fact-based arguments.

To be sure, fact and logic-focused persuasive tactics such as fact-checking are effective in the right conditions. Fact-checking is a proven technique for reducing acceptance of misinformation and disinformation [59,60,61,62] as are preemptive factual approaches, such as “pre-bunking” [63,64]. However, outside of controlled circumstances, fact-checking faces serious scalability challenges associated with widespread inadequate resources and training for digital content moderators [65,66,67]. Furthermore, fact-based approaches have proven asymmetrical, and tend to be least efficacious among the audiences most in need of intervention. Feelings of social ostracism reduce the effectiveness of fact-checking [68]. Low information audiences are less receptive to political fact-checkers, as are conservatives in general [69].

The balance of existing research tends to suggest that audiences are more likely to be persuaded by narrative approaches [70,71,72,73], appeals to values [74,75,76], and rhetorics of personal, lived experience [77,78,79,80]. Perhaps this is due to a dynamic that Maertens et al. term a “broad spectrum” of persuasion [81]. Formal dimensions of a message, such as its narrative structure, rhetorical tone, the values it prescribes, the identities to which it appeals, etc., offer more points of persuasive contact with potential audiences than the narrower logico-factual approach. However, persuasive perspectives should not be overly broad either. Mason and Miller, for example, demonstrate how negative-outcome-focused public health messages are bolstered by specific references to the manipulative strategies of (in their study) junk food marketers and advertisers [82].

Unfortunately, there is as-yet no ironclad set of optimal practices for countering misinformation and disinformation with prosocial persuasive methods. While some methods and their proper applications are more well understood, strategic efficacy still varies across real-world circumstances. A detailed understanding of the narratives and rhetorics of manipulative and damaging health misinformation and disinformation is therefore essential for governments and NGOs to remain agile and dynamic in combating anti-vaccine and COVID-denialist misinformation and disinformation. To that end, this study sought to answer the following questions:

RQ1: What are the most frequent and widely found narratives comprising francophone anti-vaccine and COVID-denialist misinformation and disinformation?

RQ2: What are the most frequent and widely found rhetorical strategies found in francophone anti-vaccine and COVID-denialist misinformation and disinformation?

RQ3: How might reception of these narratives and rhetorics vary depending on the region and culture from which their audiences originate?

## 2. Materials and Methods

This study utilizes a qualitative coding methodology to create a “codebook” of twenty-five narrative tropes and twenty-four rhetorical strategies that appeared both frequently and across a wide range of French language platforms trafficking in content deemed detrimental to COVID-related public health measures. That codebook was then analyzed by a team of experts with regional and cultural expertise in key francophone regions, who proposed regionally and culturally specific approaches to interpreting these narratives and rhetorics. This data gathering and analysis occurred between the dates of 24 March 2021 and 27 April 2021. The data sampled, extant literature, and the analysis of the regional experts point to potentially powerful approaches to crafting public health messages for linguistically unified but geographically and culturally disparate audiences.

### 2.1. Qualitative Codebooking Methodology

This study relied on qualitative coding methodology in order to empirically analyze thematic content—specifically, the narrative tropes and rhetorical strategies commonly found in online francophone anti-vaccine and COVID-denialist social media content. The findings derived from this process were then compiled into a “codebook,” consisting of codes, definitions, inclusion criteria, and examples of this content [83]. Our analysis of narrative tropes and rhetorical strategies draws from both qualitative content analysis and hermeneutical approaches to textual analysis. As in qualitative content analysis, our study followed a defined process of “selecting material; structuring and generating categories [for coding]; defining categories; revising and expanding the frame [of coding criteria]” as the central component of its analysis [84] (p. 175). Qualitative content analysis is distinct from its quantitative counterparts due to its effectiveness in pulling latent meaning from out of the text [84] In the case of this study, that latent meaning refers to the implicit narratives and manipulative rhetorical tactics that make anti-vaccine and COVID denialist media persuasive in the first place. Because this latent meaning is sometimes well below the surface of that media, our team drew on hermeneutic approaches, too, “systematically to contrast a latent and a manifest level of meaning” [85] (p. 239) and to uncover “the *latent* meaning of utterances and its relation to the intentions (manifest meaning) of actors” [85] (p. 235). Here, again, our work finds resonance with that of Stuart Hall’s theories of encoding/decoding, and the search for asymmetry between a message’s intended and overt meaning and its received and latent meaning.

This study uses collaborative analytic methods to ensure a balance of what Cornish et al. term “rich local understandings” alongside inter-coder reliability [86]. Cornish et al. explain that “local experts, as collaborators, may provide the role of a ‘guide’ or ‘educator’, explaining to the rest of the team the local context and customs—knowledge that is needed in order to produce a sensitive analysis” [86] (p. 83). Given limitations of time and budget, this rich local understanding was essential to identifying culturally specific message reception. We assembled a team of five researchers, all with full bilingual fluency in French and English, and each with domain-specific expertise in at least one region of the French-speaking world: mainland France, regions of French West Africa, the Caribbean, and North Africa. (Other francophone regions such as Quebec or Mainland Southeast Asia were not incorporated, due to staffing and budget limitations.) These researchers came from professional backgrounds ranging from doctoral studies in political science and communication, professional journalism, and NGO administration to counter-terrorism research. These researchers were supported by a team of scholars from the university who oversaw methodological training and guided the process of data identification, gathering, cleaning, coding, and final analysis.

By the same token, intercoder reliability is necessary to ensure objectivity in this interpretive work [87] (p. 344). To draw on Hall’s rubric, it was essential that coders agreed on the fundamental “hegemonic” and “professional” coding of a message, even while identifying the “negotiated” and “oppositional” readings specific to their own region and culture of expertise. Of course, an exact replication of findings is impossible in a qualitative study such as this. Instead, we adopted the standard of “inter-subjective comprehensibility” [88] (p. 187), that is, congruency between researchers’ “hegemonic” coding of the narratives and rhetorics they encountered in the dataset. Inter-subjective comprehensibility was determined using a two-step process. First, in a “single-blind” evaluation, the project manager collected researchers’ codes and compared them, evaluating similarities and grouping them according to homophily. These homophilous groupings were then named and defined so as to encompass all the codes contained under their definition. These “meta-codes” were then resubmitted to the researchers for critique, evaluation, and where necessary revision.

A more detailed description of this process is now presented below.

### 2.2. Study Design

Our team of coders underwent a three-day, twelve-hour training on the principles and practices of qualitative coding and use of the NVivo qualitative data analysis software. They were then instructed to individually seek out 5 “channels” of French-language COVID mis-, dis-, or malinformation. Channels were restricted to online social media and user generated content platforms such as Facebook, Twitter, YouTube, Telegram, and blogs. To be considered for inclusion, a channel had to meet four criteria: (1) Its content had to have been exclusively or almost exclusively French language, (2) At least 80% of the content had to have been either generally anti-vaccine, provide misinformation about COVID, or oppose common public health tactics (e.g., mask mandates), (3) It must have been updated at least once in the week prior to selection, and (4) There had to have been at least 50 pieces of relevant content posted in the last 9 months. Due to the nature of the content, many of these accounts were effectively anonymous.

Each researcher selected five channels that met this criteria, for a total of 25 possible channels. These channels were analyzed by the project manager and head of the university research team, who is French-literate, who selected three channels for a preliminary round of coding. These channels were selected based on their audience size and diversity of content. As subsequent rounds went on, channels were also selected so as to incorporate a broad range of media (text, video, meme) and platforms. The first round of coding included the Facebook page of Frank Buhler (a self-proclaimed founder of the Gillet Jeune movement), an anonymous but high-traffic Twitter account calling itself “La Croix du Sud,” and the Instagram account of Salim Laibi (a dentist who also runs the far right website *Libre Penseur*). A second round of coding included the Twitter account “Association Victimes COVID-19 France,” the Telegram channel “Le Fronte Mediatique,” and the Instagram account “La Verité Fait Mal.” The 30 most recent vaccine or COVID-specific posts from each account were scraped and coded for the narrative tropes and rhetorical strategies that researchers identified in them.

Following these two rounds of coding, the researchers delivered their codes to the project manager and university research team. These codes were refined and consolidated into a preliminary codebook, consisting of 54 narrative tropes and rhetorical strategies. This codebook was then used for a third round of coding. Channels were identified based on the same criteria as those for rounds 1 and 2; however, they were selected in hopes of finding novel codes. This meant that personal, anonymous, and more marginal sources were considered for inclusion. Round 3′s channels consisted of 10 channels: the Telegram channels “Co-Créons!!!,” “Info Vaccins France,” and “STOP Masques Vaccins,” the blog “COVIDmenace,” the Instagram accounts “DNCT.x,” and “La Verité Derange,” the Facebook account of Evelyne Wermelinger, the Twitter accounts “@Laissonslesprescire” and “Flo” (the latter selected because it appeared to be a personal account with a wide reach, which aggregated popular news stories, memes, and talking points), and a 20-min video entitled *Nouvel Ordre Mondial—Crise Coronavirus*, which was hosted on the “Hax0r” Facebook group.

Again, each member of the codebooking team separately coded 30 posts per channel, except for “COVIDmenace” (for which they coded 10 blog posts) and *Crise Coronavirus* (for which they coded the entire video). In total, round two consisted of 241 closely analyzed and coded posts. The posts were assigned to codes from the preliminary codebook created after rounds 1 and 2, and coders were given the discretion to create additional codes as they deemed necessary. After completing their analysis individually, all team members submitted their codebooks to the project manager, who again refined and consolidated their codes. It was determined that saturation had been reached, based on the diminishing number of new codes and these new codes’ marginal distinction from those of the preliminary codebook. Codes were analyzed for both the frequency with which they appeared (i.e., how many discrete posts), and the range of their appearance (i.e., across how many distinct channels). The codes with the highest numbers of raw appearances and cross-channel presence were identified. These narrative tropes and rhetorical strategies comprise the content of the final codebook.

Following the creation of the final codebook, each coding team member separately analyzed each code. Based on their cultural domain expertise, they noted which narratives and rhetorics might have culturally specific interpretations depending on the region in which they were received. Each expert offered an interpretation only for their specific region of expertise. These region- and culture-specific interpretations were then added to each code entry. A finalized codebook consisted of 23 rhetorical strategies, of which 14 were observed to yield culturally specific interpretations, and 25 narrative tropes, of which 22 yielded culturally specific interpretations. These codes will be discussed in the next section, with a focus on those culturally specific interpretations.

## 3. Results

### 3.1. Narrative Tropes

Within the 25 narrative tropes and their 22 culturally specific variation, four key tropes addressed anger and mistrust in varying cultural contexts (see Table 1, below): (i) “Caged and Enraged” (*Cage et Rage*), (ii) “The National Conspiracy” (*Le Complot National*), (iii) “Medical Dictatorship” (*Dictature Sanitaire*), (iv) “Lies and Mistrust” (*Les mensonges et la méfiance*). The data in which these narrative tropes were identified are available upon request to the corresponding author.

The narrative of “Caged and Enraged” expresses frustration at quarantine measures confining individuals to their homes, which are viewed as meaningless at best and tyrannical at worst. In the North African context, this trope might commonly be interpreted particularly through a framework of government manipulation and control. For French audiences, this code would be expected to elicit strong emotion, outrage, and implicitly encourage direct action against the measures.

The second narrative trope, “The National Conspiracy,” directly invokes distrust of government by alluding to conspiracies—both specific and vague—surrounding the vaccine and the virus itself. An example of this is the belief that there are microchips in the vaccine, or that the COVID is manmade and either designed to reduce the population or garner income for pharmaceutical companies. These narratives find footing with the rise in conspiratorial thinking in France. In West Africa, narratives fitting this category are frequently linked to fears of revenant French colonialism. In Haiti and The Antilles, where COVID rates were lower during the period of this study, these narratives corresponded with beliefs that government officials were using funds from COVID for personal gain. The North African interpretation focuses on the conspiring between the government and pharmaceutical companies to sell vaccines and control the population.

The concept of pharmaceutical industry power returns again in the narrative of “Medical Dictatorship.” Narratives belonging to this category emphasize the power of medical authority and treat safety protocols as an extension of that power and a deprivation of autonomy. Interpretations of this narrative in France draw on cultural memories of past dictatorships and invoke the Nazi occupation as an example. In North Africa, the government and medical community are viewed as working in tandem to impose control; this is comparable to the idea of “medical colonialism” that West African narratives employ.

The fourth common narrative, “Lies and Mistrust,” appeals to mistrust in the press and other official sources as well as government leaders in their reporting of and response to COVID. In France, this narrative is seen to fuel a strong pre-existing mistrust of government officials. West Africa’s relatively lower infection rate sustained this mistrust, as individuals questioned the accuracy of infection rates. Interpretations of “Lies and Mistrust” in North Africa intersect with the previously mentioned view of the government and medical community colluding in order to encourage vaccination and treatments, which (so the narrative implies) are ineffective.

### 3.2. Rhetorical Strategies

Of the 24 rhetorical strategy codes listed in this final codebook, 14 yielded culturally specific variation. Of these, four key strategies suggested certain cultural distinctions in interpretation (see Table 2, below): (i) “Concern Trolling” (*Concern Trolling*), (ii) Indignation (*Indignation*), (iii) Talking Back to Authority (*Pointer les autorités du doigt*), (iv) Minimizing/Cure is Worse than Disease (*Minimisant/le remède est pire que la maladie*). The data in which these rhetorical strategies were identified are available upon request to the corresponding author.

The rhetorical strategy of “Concern Trolling” (same in French) seeks to provoke sympathy for frontline workers and those deemed significantly affected by COVID, whether financially or mentally. However it does so for the specific purpose of denying vaccine efficacy or minimizing the severity of COVID as a public health menace. The rhetoric positions its message in a frame of selflessness, often expressing concern for those with mental or physical problems, unemployment, or depression. It uses this frame of sympathy to argue that these are problems from which COVID elimination efforts are distracting. The understanding of this strategy in West Africa challenges the Western-centered implementation of quarantine procedures that would threaten the informal markets on which many livelihoods are based and calls for regionally tailored solutions. Conversely, North African interpretations elicit an overall pessimism about society during the pandemic.

A second popular rhetoric of “Indignation” conjugates its messages in a register of annoyance and anger toward those who are pro-vaccine or following COVID protocols. This framing encourages its audience to join in ridicule of the pro-vaccine population. Interpreted in North Africa, it warns that those who follow vaccine protocols will face humiliation. In France, this code resonates with longstanding cultural values that embrace vocal expressions of indignation and outrage.

The strategy “Talking Back to Authority “preframes messages as if they were directly addressed to government officials. Messaging deploying this rhetorical strategy demands that authority reply to individual posts, which are often intoned with a sense of concern or defiance. This is seen in posts that tag government officials and seem to actually expect responses; a lack of response can be viewed as silence on the issue or cowardice on the part of the official. In France, public figures are commonly mistrusted and regularly attacked, so this strategy targets pre-existing mistrust. The West African interpretation of “Talking Back to Authority” interpellates African leaders as French puppets. In North Africa, this strategy fuels popular doubt in the authority of leaders.

A fourth popular rhetorical strategy, “Minimizing/Cure is Worse than the Disease” questions the basic legitimacy of the numbers of cases and deaths as well as the effectiveness of the vaccine. The strategy destabilizes belief in doctors and public health officials by implying that the true effects of COVID are less severe than portrayed by the media. It frames its messages around the assumption that the socio-economic costs of public health measures are underreported and underappreciated, and that these costs actually cause more harm and suffering than COVID itself. Particularly in North Africa, this rhetoric accompanies messages arguing that officials wish to generate fear and thus control the population. West African perceptions consider the fact that Africa has faced fewer cases than Europe; this is internalized by audiences as a chronic sense that official case statistics are too high to be credible.

## 4. Discussion

This study contributes to a growing body of research on the specific forms of anti-vaccine and COVID-denialist media. That body of research contributes to broader efforts at combatting mis-, dis-, and malinformation and reducing vaccine hesitancy. Members of the team responsible for this study have deployed similar study designs in the past, including one that developed an anglophone codebook of anti-vaccine and COVID-denialist narratives and rhetoric [89]. That anglophone codebook formed the basis for a series of prophylactic attitudinal inoculation videos against anti-vaccine and COVID-denialist misinformation and disinformation. These messages were demonstrated to have a significant impact in reducing perceived credibility toward the creators of anti-vaccine and COVID-denialist content [64]. It was found that fact-based inoculation messages performed equally well to narrative/rhetoric-based messages, and equally well to hybrid methods that used both fact and narrative/rhetorical techniques. It seems possible that a similar approach could be pursued to develop francophone public health messages based on persuading audiences away from anti-vaccine and COVID-denialist viewpoints. This codebook is intended to inform public health measures, from messaging campaigns to better moderation of online platforms.

## 5. Implications

This is not to say that the codebook offers no applicability on its own merits. It confirms previous studies that describe the spread of misinformation and disinformation across national boundaries via francophone networks. It demonstrates the extreme adaptability of misinformation and disinformation, even relative to changing developments in the legitimate news cycle. One may see how codes such as “The Little Guy” easily adapt to suit shifts in the population most impacted by the economic and social side-effects of necessary public health measures. Likewise, narratives such as “Medical Dictatorship” and rhetorics such as “Talking Back to Authority” are adaptable to alterations in the spokespeople explaining public health needs, or even changes of political administration. Indeed, this study’s findings indicate that facts offer limited power to impact the narratives and rhetoric that form the core of anti-vaccine and COVID-denialist mis- and disinformation. The most prevalent narratives and rhetorics are necessarily the most durable—that is, the most adaptable in responding to changing facts “on the ground.”

Unfortunately, this points to a very real challenge facing the digital media companies tasked with moderating content on their platforms. While factually incorrect information pertaining to the COVID pandemic is fairly easy to identify and remove, these persuasive narratives and rhetorics are not. Many of the most egregious narratives work via innuendo, or are hard to distinguish from hyperbole, which platforms may be reluctant to police. Rhetorics, likewise, operate in the register of emotion and affect, less concerned with rebutting facts than shaping audiences’ feelings toward those facts. To the extent that narrative and rhetoric helps to spread anti-vaccine and COVID-denialist attitudes (and that extent seems significant), they are able to spread through digital channels of news and information with only limited impediment.

This suggests a possible shift in the emphasis of news coverage may help society combat the spread of such misinformation. In addition to important updates on the latest scientific news pertaining to the COVID pandemic, mainstream, legacy, government and advocacy-based news and information sources might devote editorial space to showing how purveyors of misinformation and disinformation are adapting to these developments and incorporating them into their predetermined worldview. This is, after all, news itself in the sense of a changing development in the landscape of the pandemic. It would offer a valuable way for audiences to see anti-vaccine and COVID-denialist media’s loose and instrumentalized relationship to science, if not truth itself. While codebook-based communication such as this would not be attitudinal inoculation of the type described above, it nevertheless could play an important role in curbing the appeal of anti-vaccine and COVID-denialist propaganda.

## 6. Limitations

While this study takes advantage of its coding team’s cultural and regional expertise, it was also limited to those regions and cultures. Other francophone regions such as Quebec and Mainland Southeast Asia remained outside the scope of this study due to budgetary and staffing limitations. Unfortunately, it was also not possible to test the pervasiveness of the narratives’ and rhetorics’ culturally-specific interpretations. An expanded study might have sampled audiences from each of the regions discussed in the codebook to test the precision of the coding team’s interpretations and to identify new regionally and culturally specific interpretations. This study was also unable to perform network analysis on the flow of mis-, dis-, and malinformation through francophone social media. As per Bruns and Dotto and Cubbon, the francophone infodemic is neither one-directional nor self-contained. Anti-vaccine and COVID denialist narratives and codes presumably originate in every region of the French-speaking world and spread to every other region. Likewise, foreign misinformation and disinformation campaigns target francophone social media for a variety of reasons, from misguided to malign. It would likely have proven highly valuable to track the flow of the narratives and rhetorics listed in this study’s codebook, to better determine their origin and flows through the network.

## 7. Future Research

By pairing a follow-up francophone inoculation messaging study with the anglophone study mentioned before, we might gain greater insight into the need for audience-tailored approaches to public health messaging. Further research could indicate whether public health messages countering anti-vaccine and COVID-denialist media should be tailored for the specific regions in which they are broadcast, or if language alone is sufficient to positively influence audiences. The present study’s codebook might be used to further probe the question of fact-based vs. narrative/rhetoric-based efficacy. Given that the narratives and rhetorics identified in this study lead to different interpretations depending on their cultural reception, is it possible that fact-based prophylactic inoculation would yield overall better outcomes across pan-francophonic audiences? Or, would the so-called “blanket of immunity” effect, by which attitudinal inoculation against one manipulative persuasive technique confers resistance against similar techniques, mitigate that difference? Furthermore, research shows that generational cohort plays a role in receptivity to fake news [18]. Research into this dimension of message reception and interpretation might enrich the depth and accuracy of the study. In a situation such as the COVID pandemic, in which large numbers of people must be convinced to take a COVID vaccine, even a slight difference in message efficacy could mean the difference of many lives saved.

More abstractly, future qualitative and theoretical work might move deeper into questions unearthed by Hall’s encoding/decoding model of communication. Deeper analysis into the valences of hegemonic, negotiated, and oppositional interpretative positions across French-speaking cultures might yield important insights into preventative or counter-messaging strategies. Receptive differences between audiences in France versus in former French colonies raise tantalizing questions as to the relation between right-wing populism and the postcolony. Anti-vaccine and COVID-denialist audiences in both milieus appear to participate in oppositional decoding processes, and insofar as anti-vaccine and COVID denialism are concerned, reach similar conclusions in that oppositional decoding. Yet this dynamic seems informed by two contradictory ethos: on one hand, the right-populism of the colonizer culture appears motivated by a sense of aggrieved entitlement (resistance toward basic public health expectations) while the former colonized culture appears animated with a sense of “corrective paranoia” born of group grievance (against the perception of Western meddling in domestic affairs). This is no doubt a highly complex and fraught question, but the present study indicates that it is one with more than theoretical importance.

## 8. Conclusions

This study has identified twenty-five narrative tropes and twenty-four rhetorical strategies commonly found across a wide range of French-language anti-vaccine and COVID-denialist media. Toward the goal of answering its broad research questions, this study found the following:

RQ1: What are the most frequent and widely found narratives comprising francophone anti-vaccine and COVID-denialist misinformation and disinformation?

RQ2: What are the most frequent and widely found rhetorical strategies found in francophone anti-vaccine and COVID-denialist misinformation and disinformation?

These narratives and rhetorics represent a wide range of manipulative persuasive tactics. Most commonly, they speak to mistrust in government, social, and media authority and a belief that the costs of addressing COVID-19 as a public health matter outweigh the benefits of doing so.

RQ3: How might reception of these narratives and rhetorics vary depending on the region and culture from which their audiences originate?

Regional and culturally specific variations suggest that the breadth of their persuasive efficacy is wide indeed. These basic narratives and rhetorics often take on different meaning depending on the audience receiving them. These differences are most clearly seen in differing attitudes toward liberty and independence, divided between former colonizing and colonized nations/regions. They are also seen in regional and cultural differences relating to social censure, with different emphases placed on the negative experience of humiliation, ridicule, ostracism etc. Many key narratives and rhetorics did not suggest significant variation in cultural/regional reception, suggesting that some “hegemonic” meanings are more robust than others.

These answers raise promising possibilities as to the efficacy of wide-ranging public health messages targeting misinformation and disinformation related to the pandemic. However, it also raises questions as to underlying impetus behind these regional and cultural differences. While this study may facilitate immediate steps toward countering anti-vaccine misinformation and disinformation, it also points to important future research that ought to be undertaken.

## Figures and Tables

**Table 1:** Narratives (in English and in French).

Narratives	
**#1STWORLDPROBLEMS** The narrative that COVID panic does not exist in countries except for France and/or affluent Western nations. It suggests that concern over COVID is a bit decadent and hysterical, and developing countries are either tougher or simply don’t have the spare time to and resources to indulge in this hysteria.	**The Caribbean:** In Haiti and the Antilles, this code manifests itself with pointing to the fact that the majority of COVID cases have been contracted by people coming back from so-called developed countries and that (at the time of this study) COVID did not have a significant local source of transmission.
**Apocalypse!** Claiming that the introduction of public health measures will make a situation catastrophically worse.	**West Africa:** The vaccine is the mark of the beast. **France:** The French can have a flair for the dramatic, so this code resonates with them and acts as a call to action. France also has a strong tradition of secular public life. This rhetoric is thus less a literally religious rhetoric than a means of communicating existential nature of the struggle against public health measures.
**Caged and Enraged** The narrative that people are being needlessly confined in their houses and that they are absolutely fed up with it.	**West Africa:** Our countries and economies are not the same as the West’s. We cannot simply work from home or follow the same quarantine procedures. If we close our informal markets where the majority of people work, the population will die of hunger. We need African solutions suited to our ways of life. **North Africa:** Manipulation of the population to impose the vaccine (like the New World Order). Quarantined people and vaccinated people are in a state of distress. For example, a meme depicts Kermit the Frog wearing a mask, sitting in a pot of boiling water. This acts as a metaphor for the growing pressure on anti-maskers to conform, as well as a condemation of people going along with mask mandates, presenting them as the proverbial, insensate “boiling frog.” **France:** The French can have a flair for the dramatic, so this code resonates with them and acts as a call to action.
**The National Conspiracy** This narrative suggests that governmental and medical authorities manufactured the COVID crisis in order to control the people and make as much money as possible out of it. Typical tropes include the “Great Reset” conspiracy theory and “New World Order,” 5G, microchips in the vaccine, that COVID is a manmade creation, that the vaccine exists to reduce the population, that panic about the pandemic was created to sell more vaccines, that vaccine and vaccine passports and public health measures are conspiracies by invisible forces, that the government is in cahoots with big pharmaceutical companies, and that the vaccine was discovered suspiciously quickly.	**West Africa:** This narrative suggests that France is strengthening its colonial ties. **North Africa:** The government is conspiring with Big Pharma in order to sell as many vaccines as possible (vaccines discovered too quickly for them to be safe). Use of new censorship measures for a conspiracy to reduce the world population **France:** France, like many Western countries, has seen a resurgence in conspiratorial thinking and this is going to resonate with a certain segment of the population. **The Caribbean**: In Haiti and the Antilles, the perception is that the president is taking advantage of COVID and of the funds allocated for COVID to get rich, as during other crises, given the low rate of COVID in Haiti and the Antilles, broadly speaking.
**The Chinese conspiracy** Accusation (sometimes sincere, sometimes ironic) that China is using manipulation to (for example) step on the covidiocy accelerator…for COVID in Cambodia. Accusations of lying and conspiracy against Asian countries (the lackeys of Beijing). Directly accuses China of conspiracy and practicing a political dictatorship (the “Pekingese Joker”) and putting pressure on neighboring countries. Directly accuses Europe and China of conspiracy for pipelines that rely on the western coast of China.	**North Africa:** COVID was created in order for China to dominate its neighboring countries. **France:** These last few years, there has been a certain resistance to international/globalist pressures in France.
**Quarantining doesn’t work** The argument that quarantining doesn’t work and is not necessary to contain the pandemic.	*No significant regional/cultural variation in audience reception noted by research team.*
**The Little Guy** “Man on the street” images and interviews, to show the human effects of public health measures.	**West Africa:** The effect of the quarantine measures on market vendors who are having a hard time earning a living. **North Africa:** A call to resistance and to put a stop to submission.
**Medical dictatorship** Medical dictatorship is the concept that the state and doctors are imposing a dictatorship through measures taken against the pandemic. The medical dictatorship deprives the individual of his bodily autonomy.	**West Africa:** In other words “medical colonialism”—vaccines, aid, etcetera in exchange for neoliberalization. **North Africa:** The State and doctors are complicit in imposing the dictatorship on the population. The State is finding a reason to impose its authority on the population. The State is going so far as to deprive citizens of their bodily autonomy. Nothing will stop it. **France:** Dictatorship lives on in the consciousness of the French, and they often bring up the Nazi occupation. This code will trigger emotions on this topic.
**They’re a bunch of quacks** This suggests that the most authoritative voices in the public health crisis are also the most incompetent and make ridiculous decisions. Regardless of their prominence, importance, or legitimacy, these figures are not trustworthy, and we should stop trusting them. They do whatever they want, as if we were in the Third World.	**West Africa:** African leaders (including public health officials) amount to Western, and particularly French, puppets. In one meme, for example, a news story about an error in vaccine production in the United States is accompanied by the caption. “And we’re supposed to have confidence in them.” In West Africa, the “them” in question is multilayered: African public health officials, their French puppetmasters, and even the U.S. pharmaceutical giants who control Western leadership.
**Great awakening** This rhetoric suggests that those who resist the public health measures have woken up to the manipulation by the state and others, and that other people will slowly wake up as well. Perhaps, the narrative goes, this is a blessing in disguise; the pandemic is an eye-opening opportunity for people to finally do their own research and discover the longstanding nefariousness of the global elites, allowing them to now spread the truth and disrupt the tyranny formerly and presently inflicted upon them.	**North Africa:** Part of the population is slowly waking up to the State’s manipulation and is joining the other part that refuses to submit to it. People are resisting political leaders’ “divide and conquer” strategy of public control. **France:** France, like many Western countries, has seen a resurgence in conspiratorial thinking and this is going to resonate with a certain segment of the population.
**Dictatorial, authoritarian, or abusive government** Governmental measures against COVID-19 are an authoritarian coup. This code presents the governmental measures implemented within the context of the fight against COVID-19 as an opportunity for a group of people to exercise power without regard for laws, customs, institutions, and traditional liberties. Or, the authorities are taking advantage of the pandemic to seize power.	**West Africa:** Some of the leaders are dictators and they are using the quarantine as a tool to consolidate their tyranny. **North Africa:** In other words, you don’t have to resign yourself to being under the authority of the State. Rise up and take back your freedom. **France:** France is tired of all behavior perceived to be authoritarian **The Caribbean:** In Haiti, power is already perceived to be authoritarian and abusive. Furthermore, given the lack of COVID cases, at the time of this study that perception was not necessarily connected with COVID. However, with elections on the horizon, a resurgence in COVID cases accompanied by public health measures will be interpreted as a governmental effort to hinder democracy and install a dictatorship.
**The government is making the situation worse** All of the difficult conditions since the beginning of the public health crisis are the fault of the government and not of the coronavirus.	*No significant regional/cultural variation in audience reception noted by research team.*
**The media is the enemy** The narrative here is that the media machine, including social media, are tools of propaganda, fake news, and censorship that push the same agenda as the government, Big Pharma, or another authority figure.	**North Africa:** The State is announcing an exaggerated number of deaths in order to control the population. The government uses the media and social networks for propaganda and censorship. **France:** France, like many other Western countries, is currently seeing low levels of trust in the media.
**Lies and mistrust** Lies about COVID-19 statistics. You can’t trust the media, and you can’t trust the government, leaders, multilateral organizations, or doctors. Governments are suppressing information about other COVID-19 treatments. The government is handling the COVID response poorly, doesn’t care about us, and will use any tactic to push vaccination. There is falsification at a statistical level.	**West Africa:** Africa hasn’t been hit as hard as Europe, they’re making up the numbers because they’re too high. **North Africa:** The doctors, media, and governments are colluding so as not to care for people with effective treatments and to push them to get vaccinated with ineffective vaccines. **The Caribbean:** In the Antilles, any resurgence in COVID infections will be perceived as a machination of the government and leaders to push either a measure or the vaccine. With regard to the vaccine, residents of the Antilles allow themselves to be led by the hesitations of the rest of the world and generally want to see someone close to them get vaccinated without any side effects before they themselves will get vaccinated. **France:** The French mistrust forms of authority and will question the official statistics.
**Murderers!** The State and the doctors who impose public health restrictions are murderers because they are closing intensive care units or recommending harmful or fatal vaccines—whatever decisions they make are detrimental. A former vice-president of Pfizer has not ruled out the existence of a plan to use the vaccines for “large-scale depopulation.”	*No significant regional/cultural variation in audience reception noted by research team.*
**Nanny State** Nanny state is a government that doesn’t trust its citizens to act in their own best interests.	**France:** French culture has a deep strain of mistrust for the government. The “nanny state” concept evokes the paternalism of monarchy and clergy, against which there is a long-running opposition in French culture.
**Resistance and Liberty** This places the people in direct conflict with the State. The people are supposedly anti-restriction or anti-vax and the State is acting against the knowledge and wishes of the people who elected them. This narrative appeals to a feeling of unity with people who fight against the medical dictatorship; the movement is gaining ground and the dictatorship will tumble soon. This rhetoric suggests that civil disobedience against public health measures is a patriotic duty. This rhetoric deliberately promotes resistance to public health restrictions, showing the supposed popular resistance of the people against quarantines, masks, vaccines, and passes.	**North Africa:** The people are against all of the public health measures and they oppose the government. Some are calling for resistance and for freedom. **France:** Even in normal times, the French protest against their government often. This code will resonate with them. For example, one meme uses art evocative of WWII propaganda posters, in which a man pushes away a syringe saying “No thank-you! I am in la Résistance!”
**Defective science** This suggests that the information about the virus, tests, public health measures, vaccines, and the demands of the people are based on scientific methods that are either defective or manipulated, i.e., the tests don’t work, masks won’t protect you completely, the vaccine creates variants, etc. You shouldn’t trust the vaccine for multiple reasons. The argument that the vaccine will not work because it was developed too hastily or else because the RNA technology doesn’t work. Either that, or there are side effects (some of which we don’t yet know about) and it can kill you.	**North Africa:** Here there are a lot of doubts about the defectiveness of the science, as well as about manipulation. **The Caribbean:** In the Antilles, there are some worries about the science underlying the vaccine. More specifically, the speed with which the vaccine was produced is often pointed out as the reason for this hesitation. Furthermore, the inadequacy of the existing healthcare system is also cited as another reason for the hesitation, as serious negative side effects would amount to death.
**It’s not that big a deal** The pandemic isn’t as serious as the media or government want us to think it is. If you look at the statistics, we can see that cases are low, so nothing justifies the public health restrictions to which we are being subjected.	**West Africa:** Again, this is to say that Africans/Black people are resistant to the virus—we’re stronger than that, we’ve survived different viral epidemics before. The virus only kills white people. Africa hasn’t been hit as hard as Europe, they’re making up the numbers because they’re too high.
**Alternative treatments** The promotion of unapproved treatments to COVID-19 and/or treatment rather than vaccination. Sometimes presents religion as a protection against COVID-19. Presents an argument against vaccination and advocates the argument that everyone should be infected to achieve herd immunity (and the strongest will survive). Recommends vitamin D and zinc to treat COVID and strengthen the immune system for future cases.	**West Africa:** Turning to traditional medicine. **North Africa:** Audiences in North Africa might resonate with the axiom that “an ounce of prevention is worth a pound of cure.” That is, alternative (and dangerous/unapproved) preventative treatments like ivermectin may be appealing. **The Caribbean:** In the Antilles, natural and plant-based remedies are often extolled as a way to strengthen one’s immunity against COVID.
**Neither vaccines nor masks work** Similar to “Defective Medicine.” The argument that the vaccine will not work because it was developed too hastily or else because the RNA technology doesn’t work. This narrative suggests that there are doubts about the vaccines. It suggests that people believe vaccines to be ineffective, and that there are negative side effects (some of which we don’t yet know about) and they can kill you. Also, masks don’t work.	**France:** At the time of this study, the French were largely tired of incoherent public health policies in response to COVID-19 and have picked up the habit of categorically rejecting any new ones.
**Trauma** Interview subjects refer to soaring child abuse and levels of psychiatric trauma in children caused by COVID confinement. One interviewer likens the effects of public health measures to the after-effect of a rape.	**North Africa:** Having children wear masks is equivalent to abuse and these measures are considered ridiculous and harmful.
**Victimhood/Humiliation** A rhetoric that has the tone of being victimized, bossed around and/or humiliated	**West Africa:** Africans are victims of colonialism; they’re taking advantage of us, particularly because we’re economically/politically vulnerable to the whims of the West. **North Africa:** Victims have been pushed around, ordered around, and humiliated, and they feel discriminated against.
**We Will Not Be Silenced!** Populist appeal. The narrative of brave anti-vaxxers or anti-maskers taking great risks to spread the real truth about COVID, even as they are continually censored or threatened by the powers that be. The comparison between a mask and a muzzle suggests that any measure attempting to minimize COVID serves to silence a population that does not want all of these rules.	**North Africa:** Don’t give up your freedom and don’t submit to the restrictions of public health measures. Accusation that the government is committing crimes against humanity.
**You Can’t Stop Living Your Life** The narrative that the world shouldn’t stop for COVID and COVID shouldn’t stop you for living your life. Often used in conjunction with “It’s Not That Big A Deal” and “COVID will never die out”	**North Africa:** A call to find freedom once more and to live a normal life in coexistence with COVID-19, which becomes almost an inevitability. The State is manipulating society to better control it. But don’t underestimate the power of the mind. **The Caribbean:** In the Antilles, the people are living out this story. It will feed the opposition to any public health measure. Notably, the relative low numbers of COVID cases at the time of this study reinforced the narrative that the virus was not that big a deal.
Histoires	
**#1STWORLDPROBLEMS** Ce code démontre que le sentiment d’alerte n’existe pas qu’en France/dans les pays Francophones, alors on devrait le prendre au sérieux. Généralement, on parle des pays dits développés.	**Caraïbes:** En Haïti et dans les Antilles, ce code se manifeste en pointant vers le fait que la majorité des cas de COVID ont été contractés par des individus qui sont revenus des pays dits développés et que, à la base, le COVID n’a pas de source de transmission locale.
**L’Apocalypse** Prétendant que l’introduction des mesures imposées pour la santé publique empirera catastrophiquement la situation.	**Afrique de l’Ouest:** Le vaccin contient la marque de la bête. **France:** Les Français peuvent avoir un flair pour le dramatique, donc ce code résonne et agit comme un appel à l’action.
**Cage et rage** Histoire selon laquelle le narrateur démontre que le peuple est inutilement confiné, en ont marre et sont en détresse.	**Afrique de l’Ouest:** Nos pays et nos économies ne sont pas les mêmes que les pays Occidentaux. Nous ne pouvons pas simplement travailler à distance ou suivre les mêmes procédures de confinement. Si nous fermons nos marchés informels où la plupart des gens travaillent, la population mourra de faim. On a besoin de solutions Africaines, adaptées à nos modes de vie. **Afrique du Nord:** Manipulation de la population pour imposer le vaccin (comme le Nouvel Ordre Mondiale). Les confinés et les vaccinés sont en état de détresse. **France:** Les Français peuvent avoir un flair pour le dramatique, donc ce code résonne et agit comme un appel à l’action.
**Le Complot National** Cette narrative suggère que les autorités gouvernementales et sanitaires ont fabriqué la crise du COVID pour pouvoir contrôler le peuple ou attirer le maximjum d’argent possible de la crise. Tropes typiques à propos du COVID: «Great Reset» et «Nouvel Ordre Mondial»; 5G et puce; COVID est une création humaine; vaccin pour réduire la population. De même au sujet du vaccin: la panique est créée autour de la pandémie pour mieux vendre le vaccin. Le vaccin, les passeports sanitaires et les mesures sanitaires sont des complots organisés par des forces invisibles. Les gouvernements conspirent avec Big Pharma et la rapidité dont les vaccins ont été développés est suspecte.	**Afrique de l’Ouest:** Cette narrative suggère que la France renforce ses liens coloniaux. La perception est que le président profite de la COVID et des fonds qui ont été débloqués pour la COVID pour s’enrichir comme pendant les autres crises vu la faible présence de la COVID dan la région. **Afrique du Nord:** Conspiration des gouvernements avec Big Pharma afin de vendre le maximum possible de vaccins qui ont été développés trop rapidement pour être surs. Utilisation des nouvelles mesures sanitaites pour le complot de la réduction de la population mondiale **France:** La France, comme de nombreux pays occidentaux, connaît une résurgence de la pensée conspiratrice et cela va résonner avec un certain segment de la population. **Caraïbes:** En Haïti et dans les Antilles, encore, la perception est que le président profite de la COVID et des fonds qui ont été débloqués pour la COVID pour s’enrichir comme pendant les autres crises vu la faible présence de la COVID dan la région.
**Le Complot Chinois** Accusation (parfois sincère, parfois ironique) que la Chine utilise la manipulation pour «appuyer sur l’accélérateur de covidémentiel pour COVID au Cambodge». Accusations de mensonge et de complot contre les pays asiatiques (les laquais de Pékin). Accuse directement la Chine de conspiration et de pratique d’une dictature politique (le «farceur pékinois») et de faire pression sur les pays voisins. Accuse directement l’Europe et la Chine de complot (COVID-19) pour des pipelines qui dépendent de la côte ouest de la Chine.	**Afrique du Nord:** la COVID a été créée pour que la Chine domine les pays voisins. **France:** Ces dernières années, il y a eu une certaine résistance aux pressions internationales/mondialistes en France.
**Dictature Sanitaire** La dictature sanitaire est le concept que l’état et les médecins imposent une dictature grâce aux mesures prisent contre la pandémie. La dictature sanitaire prive l’individu de son autonomie corporelle.	**Afrique de l’Ouest:** C’est-à-dire le “colonialisme sanitaire”—vaccins, aide et cetera en échange de la néo-libéralisation. **Afrique du Nord:** La complicité de l’état et des médecins pour imposer la dictature contre les citoyens. L’état trouve une raison pour imposer son autorité contre la population. L’état va jusqu’à priver les citoyens de leurs autonomie corporelle. Rien ne l’arrête. **France:** La dictature vit dans la conscience des Français et ils évoquent souvent l’occupation nazie. Ce code déclenchera des émotions à ce sujet.
**État Nounou** L’État nounou est un gouvernement qui ne fait pas confiance à ses citoyens pour qu’ils agissent dans leur propre intérêt. Il est autoritaire, sous le déguisement de «la mère est meilleure juge.»	**France:** Les français seméfient naturellement de leur gouvernement.
**Experts Incompétents** Cela suggère que les voix les plus autoritaires dans la crise sanitaires sont aussi les plus incompétentes et prennent des décisions ridicules. Ces figures, n’importe-leur proéminence/importance/légitimité, ne sont pas fiables, et nous devons cesser de confier en eux. Ils font du n’importe quoi comme si nous étions dans le tiers-monde.	**Afrique de l’Ouest:** C’est-à-dire dirigeants africains = marionnettes françaises.
**Grand Réveil** Cette rhétorique suggère que ceux qui résistent aux mesures sanitaires se sont réveillés à la manipulation de l’État et al., et que lentement d’autres se réveillent aussi.	**Afrique du Nord:** Division de la population. On constate qu’une partie de la population se réveille lentement à la manipulation de l’état et rejoint l’autre partie qui est insoumise. **France:** La France, comme de nombreux pays occidentaux, connaît une résurgence de la pensée conspiratrice et cela va résonner avec un certain segment de la population.
**Gouvernement dictatorial, autoritaire ou abusif** Les mesures gouvernementales COVID-19 comme un coup de force autoritaire—Ce code présente les mesures gouvernementales mises en place dans le contexte de la lutte contre la COVID-19 comme une occasion pour un groupe de personnes d’exercer le pouvoir sans tenir compte des lois, des coutumes, des institutions et des libertés traditionnelles, ou même que les autorités profitent de la pandémie pour s’accaparer du pouvoir.	**Afrique de l’Ouest:** Certains dirigeants sont des dictateurs et ils utilisent le confinement comme un outil pour consolider leur tyrannie. **Afrique du Nord:** C’est-à-dire qu’il ne faut pas ce résigner a être sous l’autorité de l’état. Soulevez- vous et reprenez votre liberté. **France:** La France est lasse de tout comportement autoritaire perçu **Caraïbes:** En Haïti, le pouvoir est déjà perçu comme autoritaire et abusif. En outre, vu le manque de cas, cette perception ne s’allie pas à la COVID. Toutefois, avec les élections à l’horizon, une recrudescence de cas COVID accompagnée de mesures sanitaires restrictives sera interprétée comme un effort par le gouvernement d’entraver la démocratie et d’instaurer une dictature.
**Le gouvernement empire la situation** Toutes conditions difficiles depuis le début de la crise sanitaire est la faute du gouvernement et non du coronavirus.	*Aucune variation régionale/culturelle significative dans la réception du public notée par l’équipe de recherche.*
**Il faut vivre votre vie!** La rhétorique ici est que le monde ne peut pas et en effet ne cessera pas de tourner à cause de la pandémie alors, par conséquent, nous ne devrions pas non-plus cesser de vivre normalement. Souvent utilisé avec des codes qui suggèrent que le virus n’est pas un drame, et des codes qui suggèrent que nous devrions normaliser la coexistence avec le virus parce qu’il ne disparaîtra jamais complètement.	**Afrique du Nord:** Appel à retrouver la liberté et à vivre normalement en coexistant avec la COVID-19 qui devient presque une fatalité. L’état manipule la société pour mieux la contrôler. Mais ne sous-estime pas les esprits. **Caraïbes:** Dans les Antilles, cette histoire est vécue par la population et al.imentera l’opposition à toute mesure sanitaire. Notamment, l’absence de cas de la COVID renforce vraiment la narrative que le virus n’est pas aussi sérieux que ça.
**Médias ennemies** Le récit ici est que la machine médiatique, y compris les médias sociaux sont des outils de propagande, fake news, et de censure qui poussent le même agenda que le gouvernement/Big Pharma/autre figure autoritaire.	**Afrique du Nord:** Pour contrôler la population l’état annonce des décès exagérés. Le gouvernement utilise les médias, les réseaux sociaux pour la propagande et la censure. **France:** La France, comme de nombreux autres pays occidentaux, connaît actuellement un faible niveau de confiance dans les médias.
**Minimisant & le remède est pire que la maladie** Le récit selon lequel la pandémie de COVID n’est en réalité pas aussi grave que les médias le présentent, notamment en comparaison avec les épidémies (comme le SIDA) ou les crises passées. Big Pharma, les médecins, les responsables de la santé publique, le gouvernement, ou tous ces acteurs, exagèrent le nombre de cas et/ou de décès et la gravité de la maladie pour susciter la peur et contrôler la population. Exagérer les effets secondaires.	**Afrique de l’Ouest:** Les Africains/Noirs sont résistants au virus—nous sommes plus forts que ça, nous avons survécu différentes épidémies. Le virus ne tue que les Blancs. L’Afrique n’est pas aussi durement touchée que l’Europe, ils inventent les chiffres parce qu’ils sont trop élevés. **Afrique du Nord:** Exagération de toutes les statistiques données par toutes les autorités de la pandémie dans le but d’affoler les populations et ainsi les contrôler.
**Les mensonges et la méfiance** Il y a de la falsification et des mensonges au niveau des statistiques COVID-19; on ne peut pas faire confiance aux médias et on ne peut pas faire confiance au gouvernement, aux dirigeants, aux institutions multilatérales, ou au médecins. Les gouvernements répriment les informations sur d’autres traitements COVID-19. Le gouvernement gère mal la réponse COVID, ne se soucie pas de nous et utilisera toutes les tactiques pour pousser la vaccination.	**Afrique de l’Ouest:** L’Afrique n’est pas aussi durement touchée que l’Europe, ils inventent les chiffres parce qu’ils sont trop élevés. **Afrique du Nord:** complicité des médecins, des médias et des gouvernements pour ne pas soigner les gens avec des traitements efficaces et les pousser à se faire vacciner avec des vaccins inefficaces. **Caraïbes:** Dans les Antilles, toute recrudescence sera perçue comme une machination par le gouvernement et les dirigeants pour pousser une mesure ou le vaccin. Par rapport au vaccin, les habitants des Antilles se laissent guider par les hésitations du reste du monde et veulent généralement voir une personne de leur entourage se faire vacciner et ne pas ressentir d’effets secondaires avant de se faire vacciner eux-mêmes. **France:** Les Français se méfient des formes d’autorité et remettront en question les statistiques officielles.
**Meurtrier(e)s** L’État et les médecins qui imposent des restrictions sanitaires sont des meurtriers parce qu’ils ferment les centres de réanimation ou ils recommandent des vaccins nocifs/fatals—les décisions prises sont toujours à détriment. Un ancien vice-président de Pfizer n’exclut pas l’existence d’un projet de «dépopulation à grande échelle» par les vaccins.	*Aucune variation régionale/culturelle significative dans la réception du public notée par l’équipe de recherche.*
**Nous ne serons pas réduits au silence!** L’histoire raconté avec cet appel populiste est celle d’activistes anti-vaccins/masques courageux prenant de grands risques personnels pour parler honnêtement de la pandémie malgré les menaces et tentatives de censure. La rhétorique établie compare souvent les masques et les muselières, suggérant que toutes mesures de santé publique mises au point pour contrôler la pandémie servent à réduire au silence le peuple qui, en réalité, ne veut pas être restreint.	**Afrique du Nord:** Ne pas renoncer à la liberté et ne pas se soumettre aux restrictions des mesures sanitaires. Accusation des gouvernements de crimes contre l’humanité.
**«Le petit gars»** Interviews des personnes dans la rue ou sur la route pour démontrer l’effet humain des mesures sanitaires.	**Afrique de l’Ouest:** Effet des mesures de confinement sur les vendeurs du marché qui ont du mal à gagner un revenu. **Afrique du Nord:** Appel à la résistance et mettre fin à la soumission.
**Résistance et Liberté** Cela place le peuple en conflit direct avec l’État. Le peuple est supposément majoritairement anti-restriction/anti-vaccin et l’État agit contre les souhaits et les connaissances du peuple qui les a élus. Cela fait appel à un sentiment d’unité avec les gens qui combattent la dictature sanitaire; le mouvement gagne du terrain et la dictature va bientôt tomber. La rhétorique suggère que la désobéissance civile contre les mesures sanitaires est un devoir patriotique. Cela montre la résistance populaire supposée de la population contre le confinement/les masques/les vaccins/le laissez-passer.	**Afrique du Nord:** Les peuples sont contre toutes les mesures sanitaires et s’opposent au gouvernement. Certains appellent à la résistance et appellent à la liberté. **France:** Les Français protestent souvent contre leur gouvernement même en temps normal, ce code résonnera.
**Science défectueuse** Cela suggère que les informations sur le virus, les tests, mesures sanitaires, le vaccin, et les demandes du peuple sont basées sur des méthodes scientifiques ou bien défectueuses ou bien manipulées. i.e., les tests ne marchent pas bien, les masques ne protègent pas complètement, le vaccin crée des variantes et cetera. On ne doit pas se fier au vaccin pour de maintes raisons. Le propos est souvent que le vaccin ne marchera pas car il a été développé trop rapidement ou alors que la technologie ARN ne marche pas, ou, il a des effets secondaires (certains qu’on ne connaîtra pas maintenant) et il peut tuer.	**Afrique du Nord:** Les doutes sur la défectuosité des sciences sont très grands ainsi que ce de la manipulation. **Caraïbes:** Aux Antilles, des inquiétudes existent quant à la science qui sous-tend le vaccin. Plus précisément, la vitesse à laquelle le vaccin a été produit est souvent pointée du doigt comme la raison de cette hésitation. En outre, la faiblesse du système de santé existant est également citée comme une autre raison de l’hésitation, car des effets secondaires graves et négatifs équivaudraient à la mort.
**La situation n’est pas si grave** La pandémie n’est pas aussi grave que les médias/le gouvernement voudrat nous le faire croire. Si vous regardez les statistiques, nous pouvons voir que les cas sont faibles, donc rien ne justifie les restrictions sanitaires auxquelles nous sommes soumis.	**Afrique de l’Ouest:** C’est-à-dire que les Africains/Noirs sont résistants au virus—nous sommes plus forts que cela, nous avons survécu à différentes épidémies de virus. Le virus ne tue que les Blancs. L’Afrique n’est pas aussi durement touchée que l’Europe, ils inventent les chiffres parce qu’ils sont trop élevés.
**Traitements Alternatives & Immunité Naturelle** Promouvoir des traitements alternatifs de la COVID-19, ou traiter plutôt que vacciner. Présente parfois la religion comme protection contre le COVID-19. Utilise l’argument que l’infection de tous (et la survie des plus forts) nous permettra d’atteindre une immunité de groupe. Recommande souvent la vitamine D et le Zinc pour traiter la COVID et renforcer les défenses immunitaires des futurs infectés.	**Afrique de l’Ouest:** Recours à la médicine traditionnelle. **Afrique du Nord:** Ça vaut mieux de prévenir que guérir. **Caraïbes:** Dans les Antilles, des remèdes naturels et basés sur les plantes sont souvent prônées comme une façon de renforcer l’immunité a la COVID.
**Ni le Vaccin ni le Confinement ni le Masque Marche** Le propos que le vaccin ne marchera pas car il a été développé trop rapidement ou alors que la technologie ARN ne marche pas. Ce récit suggère qu’il y a des doutes à propos des vaccins. Cela suggère, aussi, que les gens croient les vaccins inefficaces, qu’ils ont des effets secondaires négatifs (certains qu’on ne connaîtra pas maintenant) et qu’ils peuvent tuer. Aussi, le masque ne marche pas. En fin, cela propose que le confinement ne marche pas et n’est pas nécessaire pour contenir la pandémie.	**France:** Les Français sont las des politiques de santé publique incohérentes et ont pris l’habitude d’en rejeter catégoriquement les nouvelles.
**“Tout ce que nous avons traversé n’est il pas assez?”** Le narrateur indique que le couvre-feu, la perte d’emploi, et le confinement ont déjà assez ruiné la vie des gens, et il est totalement injuste de leurs demander plus de tolérance et de soumission.	*Aucune variation régionale/culturelle significative dans la réception du public notée par l’équipe de recherche.*
**Traumatisme** Cette rhétorique suggère que les niveaux de maltraitance et de traumatisme chez les enfants en raison des mesures de confinement ont considérablement augmenté. Les conséquences des mesures de santé publique sont aussi comparées au traumatisme vécu après une agression sexuelle.	**Afrique du Nord:** Port du masque chez les enfants égale maltraitance et ces mesures sont considérées ridicules et dommageables.

**Table 2:** Rhetorical Strategies (in English and French).

Rhetorics	
**Absurdity & Ridicule** The rhetoric that the response to COVID is absurd, and implicitly, that anyone who follows these absurd public health preventative measures is a naive fool. Making fun of people who take coronavirus seriously. Has the effect of impressing that COVID is not something to take seriously. Also ridicules the “absurdity” of COVID regulations or the French government.	**West Africa:** “The virus doesn’t exist in our country. Only white people/Europeans have it in their own country, so why should we worry?” **France:** French humor is known for being dry and sharp. For example, one cartoon depicts a busker on a Métro car, announcing that he is about to perform a song. The car is packed with masked riders, who edge away horrified, as one passenger calmly explains: “If you turn this car into a theater, we will all be infected!” The serious, calm tone of the cartoon is used to ridicule what it sees as an arbitrary distinction between theater closings and ongoing Métro operation.
**ALL CAPS** Poster writers in all capital letters to indicate how dire the situation is and how vehement he is about it.	*No significant regional/cultural variation in audience reception noted by research team.*
**Freethinking authority or expert** This suggests that there are some politicians and doctors who also don’t agree with the quarantine and mask rules, and that, with enough support from the people, they could make decisions that go against the main authorities.	**West Africa:** Often using the language of “decolonization,” these “freethinkers” are congratulated for rejecting a model apparently copied from the West. **France:** The French greatly appreciate their freedom and the spirit of free thought, so they respond positively to the fact that these experts are following their own paths.
**Be Afraid … Be Very Afraid** This code uses images intended to inspire fear or anxiety in its audience. A camera slow pans in to closeups, usually of faces but sometimes of dramatic graphs, needles or other images intended to frighten, which become larger and larger in the frame and also in the imagination of the viewer.	**North Africa:** This is a denouncement of the negative authority that the State exerts over its people by relying on the fear and terror of this authority. A call to liberty against the public health measures (masks). For example, one meme shows a fit, broad-shouldered and narrow waisted young man in athletic leisure wear, pictured from the back, his hands bound by a surgical mask, evoking handcuffs. This image might remind its audience of the young men who are frequently the targets of government repression in response to political protest as, for example, during Arab Spring protests.
**Cinema Verité** Frequent shots of the camera crew as the camera pans back and forth, frequent inclusion of the mic in the shot, and even a “blooper reel” of sorts at the end. Evokes a sense of realism. Footage of either actors playing scientists or actual scientists fussing with petri dishes and syringes, in aseptically clean labs, masked and gloved; sometimes shown while the narrator asks leading questions about what might be going on in the labs.	*No significant regional/cultural variation in audience reception noted by research team.*
**Cattle, sheep, guinea pigs, animals** These comparisons either suggest that people are blindly following without thinking for themselves, and so they are easily manipulated, or that people are being experimented upon. The argument that wearing a mask is a kind of literal and figurative muzzle.	**West Africa:** Africa is not a testing site, and Africans are not guinea pigs. You are perpetuating racist colonial practices. For example, a seemingly “generic” cartoon shows a herd of sheep stampeding off a cliff. Two black sheep cry “Turn back! Something’s wrong!” A white sheep, falling off the cliff scoffs, “Another conspiracy theorist!” To a West African audience, this may evoke the dehumanizing ideology of colonization, and Western indifference to regional perspectives. **France:** The French are proud of their tradition of liberty, such that the insinuation that they are being treated like animals will resonate with them.
**Celebration** The use of emojis or language that celebrates resistance to public health measures, and that encourages others to do the same.	**France:** In France, there is a tradition of fighting against all perceived abuses of liberty, and the fighters are celebrated. The Resistance Movement during World War II.
**Concern Trolling** Rhetoric of concern for frontline workers (esp. healthcare workers) who, it is implied, are being sacrificed to the negative effects of the anti-COVID public health measures, especially the vaccine. The rhetoric of concern for the negative mental health effects of COVID, which are (it is implied) much more deleterious than the disease itself. Rhetoric of concern for elderly people and how public health measures are supposedly harming them. Tinged with reverence and pity in equal measure, intended to make the poster seem sympathetic. Rhetoric of concern for working people who lost their jobs or closed their businesses due to public health measures, framing the poster as someone sympathetic and full of solidarity for the working man. Rhetoric of concern for the effects of public health measures (particularly vaccination) on women. The narrative that any effort to eliminate COVID is somehow taking away from other, more pressing problems (i.e., AIDS, unemployment, education) Implies selflessness, objectivity, and solidarity if the speaker is a man, and solidarity/sisterhood if the speaker is a woman.	**West Africa:** Our countries and economies are not the same as the West’s. We cannot simply work from home or follow the same quarantine procedures. If we close our informal markets where the majority of people work, the population will die of hunger. We need African solutions suited to our ways of life. **North Africa:** Pessimistic opinion about the state of society during the pandemic.
**Fast-motion/Cityscapes/Ant People** Images of crowded cities full of busy people, usually masked, going quickly about their business, sometimes shown using time-lapse camera work. The impression is of the speed and bustle of people going about their lives, unaware of the big conspiracy going on behind closed doors.	*No significant regional/cultural variation in audience reception noted by research team.*
**Haven’t We Been Through Enough?** The narrative that curfews, job loss, and stay-at-home orders have already ruined the lives of the people, and that it is egregious to ask for them to tolerate them anymore	*No significant regional/cultural variation in audience reception noted by research team.*
**High-Tech** Images of glowing matrices that look like the inside of the computer or perhaps a neural network are used to introduce a profile of each new key figure, accompanied by computer-like sound effects; evocative of a “transhuman” future in which we are all part robot.	*No significant regional/cultural variation in audience reception noted by research team.*
**Hijacking Familiar Formats** Using familiar formats to convey one’s message, putting the audience at ease and priming them to be more receptive to anti-vaccine rhetoric.	*No significant regional/cultural variation in audience reception noted by research team.*
**“I’ll Just Leave This Here”** Little or no description of the content posted—sometimes just a link—indicating that the information is so obvious that theposter doesn’t need to comment on it; the reader can make up his or her own mind about it. Sometimes, this rhetoric poses a supposedly objective, innocent question—just to inform you—with answers intended to lead you to pseudoscientific conclusions.	*No significant regional/cultural variation in audience reception noted by research team.*
**Images of coronavirus** Caricatures, cartoons, photos, etc. Personified, realistic, etc.	*No significant regional/cultural variation in audience reception noted by research team.*
**Indignation** The code has a tone of indignation at the behavior of pro-vaxxers/maskers, and directly or indirectly encourages the audience to join in this indignation.	**North Africa:** The impression of humiliation for the people who want to follow public health measures or vaccination. **France:** The French are very expressive about their feelings and don’t hesitate to let others know how they feel about a situation. This code resonates with them and encourages others to express their indignation.
**Leading Questions** The rhetoric of asking questions that lead to a predetermined anti-vax answer, intended to frame the poster as calm, objective, and reasonable, and to make the audience think that they have come to their own conclusion by thinking logically and independently.	*No significant regional/cultural variation in audience reception noted by research team.*
**Manipulation** This rhetoric victimizes people and suggests that they are susceptible to the manipulation of the authorities, and so it is imperative to read information from critical voices.	**West Africa:** Not just the masses, but also our leaders are susceptible to the manipulation of France, and we are doing whatever they say without thinking for ourselves.
**Minimizing & “Cure is Worse than Disease”** The narrative that the COVID pandemic is not actually as serious as the media is presenting it to be, particularly in comparison to past epidemics (such as AIDS) or crises. Big Pharma, doctors, public health officials, the government, or all of the above, are exaggerating the numbers of cases and/or deaths and exaggerating the severity of the illness to instill fear and control the people. Exaggerating side effects. A way of delegitimizing the actions taken against the pandemic and complaining about the cost of the pandemic.	**West Africa:** Africans/Black people are resistant to the virus—we’re stronger than that, we’ve survived different viral epidemics before. The virus only kills white people. Africa hasn’t been hit as hard as Europe, they’re making up the numbers because they’re too high. **North Africa:** All of the players in the pandemic have exaggerated all the statistics they’ve given out with the goal of throwing people into a panic and thereby controlling them.
**Portmanteaus** Creation of new words, usually by joining together some form of “COVID” and an insult, i.e., “covidémence”, “couillonavirus” (in English, “COVIDiocy”, “moronavirus”); expresses poster’s above-it-all derision for the pandemic, and creating clever new lingo invites the reader to be part of the in-group that speaks it.	*No significant regional/cultural variation in audience reception noted by research team.*
**Sound the alarm!** This suggests that the message being relayed is important but also one that the authorities don’t want us to hear it. An inspirational, often upbeat rhetoric of the “little guys” taking a heroic stand against “The Man” by uniting together.	**North Africa:** Questioning the real reasons behind the quarantine, suggesting that the real reasons for the quarantine are political and no longer health-related. A meme in the dataset asks “Are we quarantined against the virus? Or against the future revolt of the people?” This again evokes long-running tensions between autocracy and popular resistance in North Africa. **France:** Any action against authority will be received well by a certain segment of the French population, as opposing authority is part of their traditional values.
**Talking Back to Authority** Messages presented as if they were speaking directly to government, health authority, the media, etc. calling out high-profile individuals by name, tagging or hashtagging them in an implied challenge for that individual to respond and defend themselves. This can have a tone of argument or even defiance. Or, messages directly addressed to authorities, tagging them on social media. Calling them out! The challenge for a reply implies that the OP and the singled-out individual are equal opponents. If the individual does not reply, the audience may interpret their silence as cowardice or a failure to come up with a defense for their actions. Often, this directly or indirectly alludes to President Emmanuel Macron, implying that he is to blame for absurd, ineffective, or intrusive measures.	**North Africa:** They doubt the transparency of the people in charge and think that they are hiding the truth. **West Africa:** In other words, African leaders = French puppets. **France:** The French particularly mistrust authority, and public figures are regularly reprimanded and attacked even in normal times.
**Unflattering Photo** The poster chooses a particularly unflattering photo of their pro-vax/pro-mask subject in an effort to make the audience instinctively dislike them.	**North Africa:** Before imposing strict measures on the population, people in charge need to respect the people imposing them. Unflattering photos promote an easy path to disrespect. **France:** In France, criticizing authority figures by any means necessary has long been a national practice.
**Legitimate voice** This suggests that the person or publication sounding the alarm is trustworthy. Often for publications, one doesn’t see the contents of the article, just the title.	**West Africa:** As with “Freethinking Authority or Expert,” this employs the language of “decolonization,” and these freethinkers are congratulated for rejecting a model apparently copied from the West. The commentator is often an economist/religious figure.
**Victimization** The practice of painting oneself as a victim of a State that abuses coronavirus restrictions.	**West Africa:** Africans are victims of colonialism; they’re taking advantage of us, particularly because we’re economically/politically vulnerable to the whims of the West. **North Africa:** Victims have been pushed around, ordered around, and humiliated, and they feel discriminated against
Rhétorique	
**Absurdité et ridicule** Insinue que la réponse au COVID est absurde, et implicitement, que ceux qui suivent ces mesures sont des imbéciles et sont naïfs. Le code se moque des gens qui prennent au sérieux le coronavirus et a pour effet de suggérer que la COVID n’est pas quelque chose à prendre au sérieux. Ça ridiculise aussi le gouvernement français.	**Afrique de l’Ouest:** «Le virus n’existe pas dans notre pays. Seuls les Blancs/Européens l’ont dans leurs propres pays, alors pourquoi est-ce qu’on s’inquiète ici ? **France:** L’humour français est réputé pour être sec et perçant, donc les Français seront sûrement sensibles à ce code.
**Action Rapide/Paysages Urbains/Personnes Occupées** Des images de villes bondées de gens affairés et généralement masqués, qui vaquent rapidement à leurs occupations, parfois à l’aide d’une caméra en accéléré. L’impression est celle de la vitesse et de l’agitation des gens qui s’occupent de leurs affaires sans se rendre compte de la grande conspiration qui se déroule derrière les portes fermés.	*Aucune variation régionale/culturelle significative dans la réception du public notée par l’équipe de recherche.*
**Autorité ou expert libre penseur** Cela suggère qu’il y a quelques politiciens/médecins qui, eux aussi, ne sont pas d’accord avec les règles du confinement/port du masque et cetera, et qu’avec suffisamment de soutien du peuple, ils/elles pourront prendre des décisions qui vont contre les autorités centrales.	**Afrique de l’Ouest:** Utilisant souvent le langage de la «décolonisation», ces libres penseurs sont souvent félicités pour avoir rejeté le modèle apparemment copié de l’Occident. **France:** Les Français apprécient beaucoup leurs liberté et l’esprit de libre-pensée, ils répondraient donc positivement au fait que les experts suivent leurs propres chemins.
**Ayez peur ⋯ ayez très peur!** Le code utilise des images destinées à inspirer la peur ou l’anxiété à son public. La caméra effectue des panoramiques lents sur des gros plans, généralement de visages, mais parfois de graphiques dramatiques comme des aiguilles, ou d’autres images destinées à effrayer, qui deviennent de plus en plus grandes dans le cadre et dans l’imagination du spectateur.	**Afrique du Nord:** C’est une dénonciation de l’autorité négative qu’exerce l’état sur les populations en s’appuyant sur les faits de peur et de terreur. Appel à la liberté contre les mesures sanitaires (masque).
**Bétail, Mouton, Cobaye, Animal** Ces comparaisons suggèrent que le peuple suit sans penser pour lui-même et donc est facilement manipulé, ou bien qu’il sert d’experiment. Le propos est que le port du masque est une sorte de “muselière,” littéralement et figurativement.	**Afrique de l’Ouest:** L’Afrique n’est pas un terrain d’essai, les Africains ne sont pas des cobayes. Vous perpétuez des pratiques coloniales et racistes. **France:** Les Français sont fiers de leur tradition de liberté, de sorte que l’insinuation qu’ils sont traités comme des animaux résonnera.
**Célébration** L’usage d’émoticônes/langages qui célèbrent la résistance aux mesures sanitaires, et qui encourage les autres à faire la même chose.	**Afrique du Nord:** Dénonciation du manque d’égalité au états, unis au nom du respect des libertés. **France:** En France, il existe une tradition de lutte contre tout abus de liberté perçu, et les combattants sont célébrés, comparable au mouvement de la Résistance pendant la Seconde Guerre Mondiale.
**“Concern Trolling”** Une rhétorique de préoccupation pour les travailleurs de première ligne, les personnes âgées, les chômeurs, les femmes et les enfants, entre autres. Le sousentendu est que les effets secondaires des mesures sanitaires, comme le chômage, les maladies mentales, la violence familiale et cetera, sont pires que la pandémie en elle-même. Implique un esprit de solidarité pour ceux qui souffrent le plus des confinements et couvre feux et cetera.	**Afrique de l’Ouest:** Nos pays et nos économies ne sont pas les mêmes que les pays Occidentaux. Nous ne pouvons pas simplement travailler à distance ou suivre les mêmes procédures de confinement. Si nous fermons nos marchés informels où la plupart des gens travaillent, la population mourra de faim. On a besoin de solutions Africaines, adaptées à nos modes de vie. **Afrique du Nord:** Avis pessimiste sur l’état de la société durant la pandémie.
**Détournement de formats familiers** Utilisation de formats familiers pour transmettre un message en mettant le lecteur à l’aise et le rendre plus réceptif à la rhétorique anti-vaccin.	*Aucune variation régionale/culturelle significative dans la réception du public notée par l’équipe de recherche.*
**Haute Technologie** Des images de matrices lumineuses qui ressemblent à l’intérieur d’un ordinateur ou peut-être à un réseau neuronal, utilisées pour introduire le profil de chaque nouveau personnage clé, accompagné d’effets sonores dignes d’un ordinateur; elles évoquent un avenir “transhumain” dans lequel nous sommes tous en partie des robots.	*Aucune variation régionale/culturelle significative dans la réception du public notée par l’équipe de recherche.*
**Images du Coronavirus** Caricature, bande dessinée, photographie, et cetera du coronavirus personnifié ou réaliste.	*Aucune variation régionale/culturelle significative dans la réception du public notée par l’équipe de recherche.*
**Indignation** Le code a un ton d’indignation au sujet des comportements des gens pro-vaccins/masques, il invite et encourage le public à joindre dans leur indignation.	**Afrique du Nord:** Impression d’humiliation pour les personnes qui veulent suivre les mesures sanitaires ou la vaccination. **France:** Les Français sont expressifs de leurs sentiments et n’hésitent pas à faire savoir aux autres ce qu’ils ressentent face à une situation. Ce code résonne et encourage les autres à exprimer leur indignation.
**“Je vais juste laisser ça ici…”** Peu ou pas de description du contenu posté—parfois juste un lien—indiquant que l’information est si évidente que le publicateur original n’a pas besoin de la commenter. Le lecteur peut former son propre avis à ce sujet. Quelques fois, cette forme rhétorique pose des questions supposément objectives et innocentes dans le but de «simplement informer». Les réponses aux questions visent à inciter au lecteur/auditeur de faire des conclusions pseudoscientifiques.	*Aucune variation régionale/culturelle significative dans la réception du public notée par l’équipe de recherche.*
**Manipulation** Cette rhétorique fait une victime du peuple et suggère qu’il est susceptible à la manipulation des autorités alors il est indispensable de lire des informations qui viennent de voix critiques.	**Afrique de l’Ouest:** Non seulement les masses, mais nos dirigeants sont susceptibles à la manipulation de la France et nous faisons tout ce qu’ils font ou disent sans penser par nous-mêmes.
**Pointer les autorités du doigt** Des messages adressés directement aux gouvernements, autorités sanitaires, médias, et cetera, soit en les «taguant» dans le message ou en plaçant un hashtag devant leur nom, en direct défi. Une forme de défiance et argumentation. Le format positionne les deux individus, la personne qui nomme et la personne nommée, comme des adversaires égaux. Si la personne ne répond pas, le public peut interpréter son silence comme de la lâcheté, ou une incapacité de défendre leurs actions. Fréquemment, cette rhétorique vise directement ou indirectement le Président Emmanuel Macron, ce qui implique qu’il est responsable pour les mesures absurdes, inefficaces ou intrusives.	**Afrique du Nord:** On doute de la transparence des responsables et on pense qu’ils cachent la vérité. **Afrique de l’Ouest:** C’est-à-dire, dirigeants africains = marionnettes françaises. **France:** Les Français se méfient particulièrement de l’autorité et les personnalités publiques sont régulièrement réprimandées et attaquées, même en temps normal.
**Photo peu flatteuse** L’affiche choisit une photo particulièrement peu flatteuse de son sujet pro-vaccin/pro-masque dans le but de susciter l’aversion instinctive du public.	**Afrique du Nord:** Avant d’imposer des mesures strictes sur la population il faut que les responsables respectent des autorités. **France:** En France, critiquer les autorités par tous les moyens nécessaires a toujours été un comportement national.
**Portemanteaux** Création de nouveaux mots en joignant «COVID» et des mots insultants pour exprimer la dérision envers la pandémie et les mesures sanitaires. La familiarité avec ces portemanteaux invitent les lecteurs à faire partie d’une communauté qui ne prend pas la pandémie au sérieux. Exemples: «covidémence», «couillonavirus» (en anglais, «COVIDiocy», «moronavirus»	*Aucune variation régionale/culturelle significative dans la réception du public notée par l’équipe de recherche.*
**Questions suggestives** La rhétorique consiste de poser des questions qui mènent à une réponse anti-vaccin prédéterminée, dans le but de positionner le publicateur comme personne calme, objective et raisonnable. Elle fait aussi croire au public que le publicateur est arrivé à sa propre conclusion en réfléchissant de manière logique et indépendante.	*Aucune variation régionale/culturelle significative dans la réception du public notée par l’équipe de recherche.*
**Sonne l’alerte** Cela suggère que le message relayé est un message important mais un message que les autorités ne veulent pas que nous ayons.	**Afrique du Nord:** Questionnement des vraies raisons derrière le confinement, ce qui suggère que les vraies raisons du confinement sont politiques et non sanitaires. **France:** Toute action contre les autorités sera bien accueillie au sein d;un certain segment de la population française car s;opposer aux autorités fait partie des valeurs traditionelles.
**Tout en majuscules** Des affiches écrites en lettres majuscules pour indiquer le grave niveau de la situation et le point auquel le publicateur est concerné.	*Aucune variation régionale/culturelle significative dans la réception du public notée par l’équipe de recherche.*
**Voix Légitime** Cela suggère que la position de la personne/publication qui sonne l’alarme est fiable. Souvent, pour les publications on ne voit pas le contenu de l’article, juste le titre.	**Afrique de l’Ouest:** Utilisant souvent le langage de la «décolonisation», ces libres penseurs sont souvent félicités pour avoir rejeté le modèle apparemment copié de l’Occident. Souvent un commentateur économiste ou une figure religieuse.
**Victime et Humilié(e)** Le ton de cette rhétorique suggère que l’interlocuteur est une victime, qu’il ou elle se fait bousculer et ordonner, et qu’il ou elle se sent humilié(e) en tant que victime d’un état qui abuse les restrictions sanitaires.	**Afrique de l’Ouest:** Les Africains sont des victimes coloniales, on se profite de nous, en particulier parce que nous sommes économiquement et politiquement vulnérables aux caprices de l’Occident. **Afrique du Nord:** Les victimes se font bousculer, humilier, et ordonner et se sentent discriminés.

## Data Availability

Data is available upon request to the corresponding author.
